# Microbiome Signatures in a Fast- and Slow-Progressing Gastric Cancer Murine Model and Their Contribution to Gastric Carcinogenesis

**DOI:** 10.3390/microorganisms9010189

**Published:** 2021-01-17

**Authors:** Prerna Bali, Joanna Coker, Ivonne Lozano-Pope, Karsten Zengler, Marygorret Obonyo

**Affiliations:** 1 Department of Medicine, School of Medicine, University of California, San Diego, La Jolla, CA 92093, USA; pbali@health.ucsd.edu (P.B.); ilozanopope@ucsd.edu (I.L.-P.); 2 Department of Pediatrics, University of California, San Diego, La Jolla, CA 92093, USA; jkcoker@health.ucsd.edu (J.C.); kzengler@ucsd.edu (K.Z.); 3 Center for Microbiome Innovation, University of California, San Diego, La Jolla, CA 92093, USA; 4 Department of Bioengineering, University of California, San Diego, La Jolla, CA 92093, USA

**Keywords:** *Helicobacter*, microbiome, gastric cancer, MyD88, Lactobacillales, TRIF

## Abstract

Gastric cancer is the third most common cause of death from cancer in the world and infection with *Helicobacter* *pylori* (*H. pylori*) is the main cause of gastric cancer. In addition to *Helicobacter* infection, the overall stomach microbiota has recently emerged as a potential factor in gastric cancer progression. Previously we had established that mice deficient in myeloid differentiation primary response gene 88 (MyD88, *Myd88^−/−^*) rapidly progressed to neoplasia when infected with *H. felis*. Thus, in order to assess the role of the microbiota in this fast-progressing gastric cancer model we investigated changes of the gastric microbiome in mice with different genotypic backgrounds: wild type (WT), MyD88-deficient (*Myd88^−/−^*), mice deficient in the Toll/interleukin-1 receptor (TIR) domain-containing adaptor-inducing interferon-β (TRIF, *Trif*^Lps2^), and MyD88- and TRIF-deficient (*Myd88^−/−^*/*Trif*^Lps2^, double knockout (DKO)) mice. We compared changes in alpha diversity, beta diversity, relative abundance, and log-fold differential of relative abundance ratios in uninfected and *Helicobacter* infected mice and studied their correlations with disease progression to gastric cancer *in situ*. We observed an overall reduction in microbial diversity post-infection with *H. felis* across all genotypes. Campylobacterales were observed in all infected mice, with marked reduction in abundance at 3 and 6 months in *Myd88^−/−^* mice. A sharp increase in Lactobacillales in infected *Myd88^−/−^* and DKO mice at 3 and 6 months was observed as compared to *Trif*^Lps2^ and WT mice, hinting at a possible role of these bacteria in gastric cancer progression. This was further reinforced upon comparison of Lactobacillales log-fold differentials with histological data, indicating that Lactobacillales are closely associated with *Helicobacter* infection and gastric cancer progression. Our study suggests that differences in genotypes could influence the stomach microbiome and make it more susceptible to the development of gastric cancer upon *Helicobacter* infection. Additionally, increase in Lactobacillales could contribute to faster development of gastric cancer and might serve as a potential biomarker for the fast progressing form of gastric cancer.

## 1. Introduction

Gastric cancer is the sixth most common cancer and third most common cause of cancer mortality in the world [1]. By far the largest risk factor for gastric cancer development is the presence of the carcinogenic microbe *Helicobacter pylori* [2]. Infection with *H. pylori* leads to development of premalignant lesions that progress from gastric atrophy to metaplasia, dysplasia, and finally to gastric adenocarcinoma. While *H. pylori* infects almost 50% of the global population, only 1–3% of the infected individuals develop gastric cancer [3]. Several other factors contribute to gastric cancer progression, such as *Helicobacter* strains, environmental factors, external factors like alcohol consumption [4] and host immune response. In addition, the microbiota of the stomach may also influence final disease outcome [3]. Recently, studies have revealed that thrombosis also can be one of the reasons for fatality in gastric cancer patients [5].

The stomach had been traditionally considered a sterile organ due to its highly acidic environment and digestive juices. It was only after the discovery of *H. pylori* in 1982, and its ability to survive in such harsh conditions, that this led to the idea of a gastric microbiome, similar to microbial communities inhabiting other parts of the human host. Advancement in DNA sequencing strategies and computational methods have uncovered a complex microbiome of the stomach [3]. The gastric microbial density is estimated to be 10^2^–10^4^ colony forming units (CFU)/mL, a much lower density than other body sites, such as the colon, which often reaches 10^10^–10^12^ CFU/mL [6]. Early characterization of the gastric microbiota relied on culturing techniques but the advancement of sequencing techniques has led to the identification of different species falling into five predominant phyla—Firmicutes, Proteobacteria, Actinobacteria, Bacteroidetes and Fusobacteria, indicating a distinct microbiome of the stomach [6,7]. Thus, with the presence of such complex microbial diversity in the stomach, interactions between *Helicobacter* and other gut bacteria could potentially play a role in deciding the fate of gastric cancer progression, or may act as an aide along with other factors in driving the disease.

*H. felis* is a close relative of human gastric pathogen *H. pylori* and studies have shown that *H. felis* infection leads to the development of high-grade dysplastic lesions in C57BL/6 mice as compared to *H. pylori*, mimicking gastric carcinogenesis as seen in human [8,9,10,11]. Moreover, we have previously shown that Myeloid differentiation primary response gene 88 (MyD88) regulates *Helicobacter* induced gastric cancer progression, where MyD88 deficient mice (*Myd88^−/−^)* infected with *H. felis* showed fast progression to gastric cancer in situ as compared to wild-type (WT) mice proving it to be a better *Helicobacter* model in rodents [8]. Furthermore, we also observed that cells from mice deficient in MyD88 showed increased secretion of interferon (IFN)-α after *H. pylori* infection suggesting MyD88-independent induction of type I IFNs, suggesting role of Toll/IL-1R (TIR) domain-containing adaptor-inducing interferon-β (TRIF)-dependent pathway [12]. This prompted us to study the microbial diversity of the stomach in mice with different genotypic backgrounds: Wild type (WT), MyD88 deficient (*Myd88^−/−^*), TRIF (*Trif*^Lps2^), and MyD88 and TRIF deficient (*Myd88^−/−^*/*Trif*^Lps2^, double knockout (DKO)) mice.

Given the potential connection between the gastric microbiome and cancer progression, we hypothesized that the fast-progressing *Myd88*^−/−^ cancer model would show distinct gastric microbiome changes compared to the other genotypes. Gastric microbiome diversity and composition was, therefore, studied both in uninfected and *H. felis* infected mice of all four genotypes. In addition, we also investigated whether the gastric microbiome changed over time in response to infection and studied the correlation of these changes with gastric cancer progression. Therefore, this study would aid in identifying microbial species whose abundance or scarcity may contribute to progression of *Helicobacter*-induced lesions and towards adenocarcinoma. Ultimately, identification of other contributing factors would open new avenues of research in gastric cancer disease progression.

## 2. Material and Methods

### 2.1. Animals

Six- to 10-week-old wild-type (WT) (*n* = 42), MyD88 deficient (*Myd88^−/−^*) (*n* = 47), TRIF deficient (*Trif*^Lps2^) (*n* = 46), and double knockout (*Myd88^−/−^*/*Trif*^Lps2^, DKO) (*n* = 37) mice in the C57BL/6 background were used in this study. WT mice were purchased from The Jackson Laboratory (Bar Harbor, ME, USA). *Myd88^−/−^*, *Trif*^Lps2^, and DKO mice were from our breeding colony originally provided by Dr. Akira (Osaka University, Japan) and backcrossed 10 times onto a C57BL/6 background, bred, and maintained at University of California, San Diego (UCSD). All mice were housed together before infection with *H. felis* and for the duration of the study for each genotype. The institutional Animal Care and Use Committee at the University of California, San Diego, approved all animal procedures and they were performed using accepted veterinary standards.

### 2.2. Bacterial Growth Conditions

*Helicobacter felis*, strain CS1 (ATCC 49179) was purchased from the American Type Culture Collection (Manassas, VA, USA). *H. felis* was routinely maintained on solid medium, Columbia agar (Becton Dickinson, MD, USA) supplemented with 5% laked blood under microaerophilic conditions (5% O_2_, 10% CO_2_, 85% N_2_) at 37 °C and passaged every 2–3 days as described previously [8,12,13]. Prior to mouse infections, *H. felis* was cultured in liquid medium, brain heart infusion broth (BHI, Becton Dickinson, MD, USA) supplemented with 10% fetal calf serum and incubated at 37 °C under microaerophilic conditions for 48 h. Spiral bacteria were enumerated using a Petroff-Hausser chamber before infections.

### 2.3. Mouse Infections

This study used a well-characterized cancer mouse model, which involves infecting C57BL/6 mice with *H. felis* (strain CS1), a close relative of the human gastric pathogen *H. pylori*. Mice were inoculated with 10^9^ organisms in 300 μL of BHI by oral gavage three times at 2-day intervals as previously described [8,12]. Control mice received BHI only. At 1 month, 3 months, and 6 months post-infection, mice were euthanized, and the stomachs removed under aseptic conditions. The stomach was cut longitudinally and tissue sections were processed for DNA extraction and histopathology.

### 2.4. Histology

Longitudinal sections of stomach tissue from each mouse were fixed in neutral buffered 10% formalin and embedded in paraffin, and 5 µm sections were stained with hematoxylin and eosin (H&E). Gastric histopathology mucous metaplasia was scored by a blinded comparative pathologist (Rickman) using criteria developed by Rogers et al. [14]. Scores ranging from 0 (no lesions) to 4 (severe lesions) were measured in increments of 0.5, as previously described (9).

### 2.5. DNA Extraction

DNA was extracted from gastric tissue obtained from *H. felis*-infected and uninfected WT, *Myd88^−/−^*, *Trif*^Lps2^, and DKO mice. Stomach tissue sections were analyzed at different time points of 1 month, 3 months, and 6 months. DNA was extracted using DNAeasy Blood and Tissue kit (Qiagen, MD, USA) following manufacturer’s instructions. The DNA concentration was quantified using NanoDrop 1000 Spectrophotometer (Thermo Fisher Scientific, Waltham, MA, USA) before proceeding to DNA Sequencing.

### 2.6. 16. S rRNA Gene Sequencing

Purified DNA was amplified and processed according to Earth Microbiome Project (EMP) standard protocols (https://www.earthmicrobiome.org/protocols-and-standards/16s/) Amplicon polymerase chain reaction (PCR) was performed on the V4 region of the 16S rRNA gene using the primer pair 515F/806R with Golay error-correcting barcodes on the reverse primer. 240 nanograms of each amplicon was pooled and purified with the MoBio UltraClean PCR cleanup kit (Qiagen, MD, USA) and sequenced on the Illumina MiSeq sequencing platform. Demultiplexed FASTQ files were processed using Quantitative Insights into Microbial Ecology (QIIME2) version 2019.10 (https://qiime2.org) [15]. Sequences were denoised using Deblur (default settings, --p-trim-length 150) [16]. Taxonomy was assigned using SATé-enabled phylogenetic placement (SEPP) fragment insertion with a classifier trained on the Greengenes13_8 99% operational taxonomic units (OTUs) dataset, with sequences trimmed to contain 150 bases from the region amplified in sequencing [17]. All reads assigned to the phylum *Cyanobacteria* were filtered from the counts table before analysis, as this is considered a food contaminant in gut microbiome sequencing. A pseudocount of 1 was added to the final counts table in Songbird analysis to prevent samples dropping out of the log fold-differential analysis due to inability to calculate the logarithm of 0. FASTQ files can be found through the NCBI BioSample database (accession number PRJNA685500). Code used to process sequencing and conduct bioinformatics processing can be found on Github (https://github.com/jkccoker/Murine_gastric_microbiome).

### 2.7. Bioinformatics Processing

Taxonomy abundance plots were generated using the PhyloSeq package [18]. Taxonomy was collapsed to the order of interest in PhyloSeq. Alpha diversity plots were generated in R using data exported from the QIIME2 analysis (--p-sampling-depth 2300). Beta diversity principal component analysis plots were generated in QIIME2 with the DEICODE plug-in [19]. Taxonomy abundance log-fold differentials were calculated through QIIME2 using the Songbird plug-in (--p-formula “Genotype + Infection + Time”) [20], with visualization through Qurro (intercept = “Infection: Yes”) [21]. Receiver operating characteristic (ROC) curves were generated in Prism 7 (GraphPad Software, San Diego, CA, USA) using log-fold differential values from the Songbird analysis.

### 2.8. Statistics

Differences in alpha diversity and log-fold differentials were calculated in R using Student’s *t*-test, analysis of variance (ANOVA) or the Kruskal-Wallis H test, where appropriate. Data were checked for normal distribution before the Student’s *t*-test and ANOVA. Differences in beta diversity were assessed with permutational multivariate analysis of variance (PERMANOVA) in QIIME2 with Benjamini-Hochberg false discovery rate (FDR) correction. Ordinal logistic regression was conducted in R using the polr command (formula = histology_score ~ <log-fold differential>, Hess = T).

## 3. Results

### 3.1. Gastric Mucosal Microbial Diversity Varies with Infection Status and Genotype

**Figure 1:**
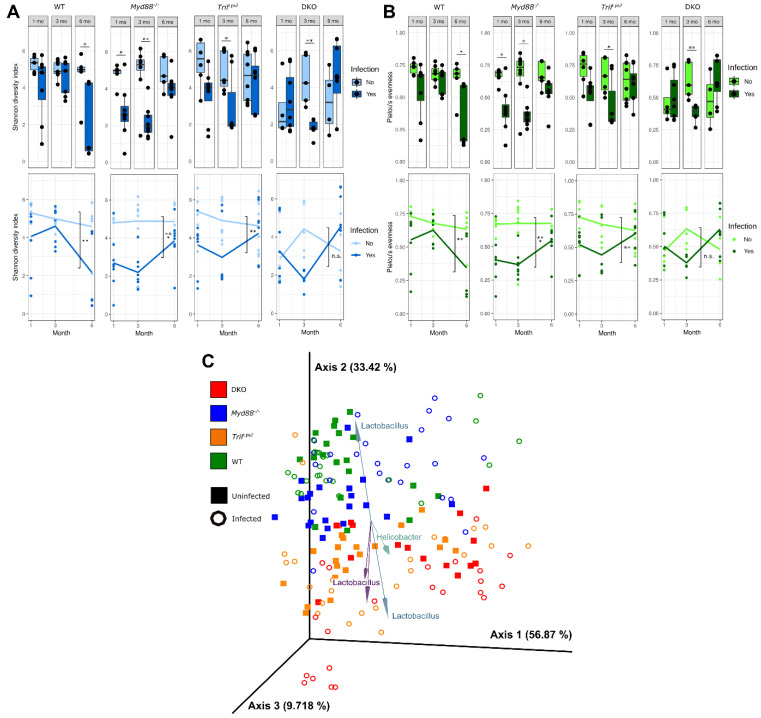
Changes in microbial diversity in the stomach across four different genotypes, with infection and time. Shannon diversity index (**A**) and Pielou’s evenness (**B**) values for each gastric community, divided by genotype and month. Top and bottom plots represent the same data, alpha diversity values, with lines on the bottom plots denoting average values for infected and uninfected communities over time. Statistical significance on bottom plots refers to differences between infected and uninfected, months 1–6 combined. (**C**) Principal component analysis of robust Aitchison distance values between communities, months 1–6 combined. Biplot arrows indicate operational taxonomic units (OTUs) driving separation between samples, with arrows labeled with the genus of the OTU. Arrows and genus labels are matched by color. All diversity metrics were calculated using QIIME2. Statistical significance determined by Student’s *t*-test for alpha diversity and permutational multivariate analysis of variance (PERMANOVA) with Benjamini-Hochberg FDR correction for beta diversity (* *p* < 0.05, ** *p* < 0.01, *** *p* < 0.005).

To assess the diversity of the gastric microbial community in each mouse genotype, we first determined alpha and beta diversity. The Shannon diversity index and Pielou’s evenness score were calculated as metrics of alpha diversity. The Shannon diversity index provides a metric of community diversity based on the number of taxa present and the abundance of each species. Differences in genotype did not significantly affect Shannon diversity index (Figure 1A). In general, infection with *H. felis* resulted in a decreased diversity index (statistically significant for WT at 6 months (*p* < 0.05); *Myd88^−/−^* at 1 month (*p* < 0.05) and 3 months (*p* < 0.01); *Trif*^Lps2^ at 3 months (*p* < 0.05); and DKO at 3 months (*p* < 0.01). For the three knockout genotypes, diversity was decreased in infected samples at 1 and 3 months but increased back to uninfected levels at 6 months. The opposite was observed in the WT genotype. Using Pielou’s evenness, which assesses how evenly distributed taxa are within a community, the evenness scores followed the same trends as the Shannon diversity index, as expected (Figure 1B).

To assess the dissimilarity between the gastric microbial communities, we next analyzed the robust Aitchison distance between communities as a metric of beta diversity (Figure 1C). Aitchison distance is a compositional metric of the Euclidean distance between samples after centered log-ratio transformation [22]. Robust Aitchison distance analysis incorporates matrix completion to account for the large number of zeros in microbiome data sets due to the absence of individual taxa in samples [19]. PERMANOVA analysis of the robust Aitchison distance between samples showed significant separation between communities by all genotype pairs (*p* < 0.001) except WT and *Myd88^−/−^* (*p* = 0.20). Pairwise comparison (with Benjamini–Hochberg FDR correction) showed that infection groups within each genotype were significantly different from other groups (*p* < 0.05), with the exception of the following interesting pairs: WT uninfected/infected (*p* = 0.07); *Myd88^−/−^* uninfected/infected (*p* = 0.07); WT infected/*Myd88^−/−^* infected (*p* = 0.15); *Trif*^Lps2^ infected/DKO uninfected (*p* = 0.42); and *Trif*^Lps2^ infected/DKO infected (*p* = 0.05). Visualization of principal coordinate analysis (PCA) displayed that WT/*Myd88^−/−^* genotypes and *Trif* ^Lps2^/DKO genotypes had more similar gastric communities, despite statistically significant differences (Figure 1C). A biplot of the robust Aitchison PCA allowed us to visualize the OTUs most heavily influencing community dissimilarity as vectors in the PCA axes (Figure 1C, arrows). These vectors revealed that the operational taxonomic units most heavily influencing the distance between samples were from the *Helicobacter* and *Lactobacillus* genera.

### 3.2. Variation in Abundance of Microbial Taxa in Different Genotypes over Time and after H. Felis Infections

**Figure 2:**
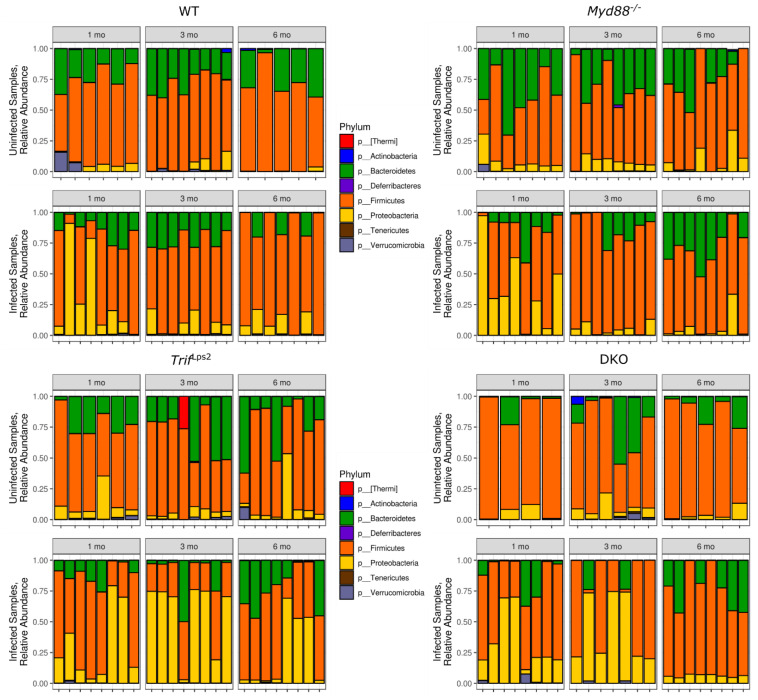
Relative abundance of different phyla across four genotypes. Relative abundance of individual phyla from WT, *Myd88^−/−^, Trif*^Lps2^, and double knockout (DKO) genotypes. The top eight phyla are shown in the legend. Samples are grouped into *Helicobacter*-infected and non-*Helicobacter*-infected and divided by time point. Sequencing data were processed in QIIME2, then plotted in PhyloSeq.

Given the differences in community diversity between genotypes and infection status, we conducted taxonomic analysis of the 16S rRNA gene sequences in each sample. The predominant phyla across all genotypes, irrespective of infection status and time included, *Bacteroidetes*, *Firmicutes* and *Proteobacteria* (Figure 2). *Bacteroidetes* and *Firmicutes* were present both in uninfected and infected samples with insignificant variations between genotypes. *Proteobacteria* were observed in high abundance in infected samples as compared to uninfected samples in all genotypes as expected (Figure S1). However, the levels of *Proteobacteria* dropped significantly at 3 months and 6 months in infected *Myd88*^−/−^ mice and at 6 months for DKO mice in infected samples. In contrast, in *Trif*^Lps2^ and WT no such drop was observed. Moreover, the levels of *Proteobacteria* in *Trif*^Lps2^ were similar at 1 month and 6 months, while peaking at 3 months (Figure S1).

**Figure 3:**
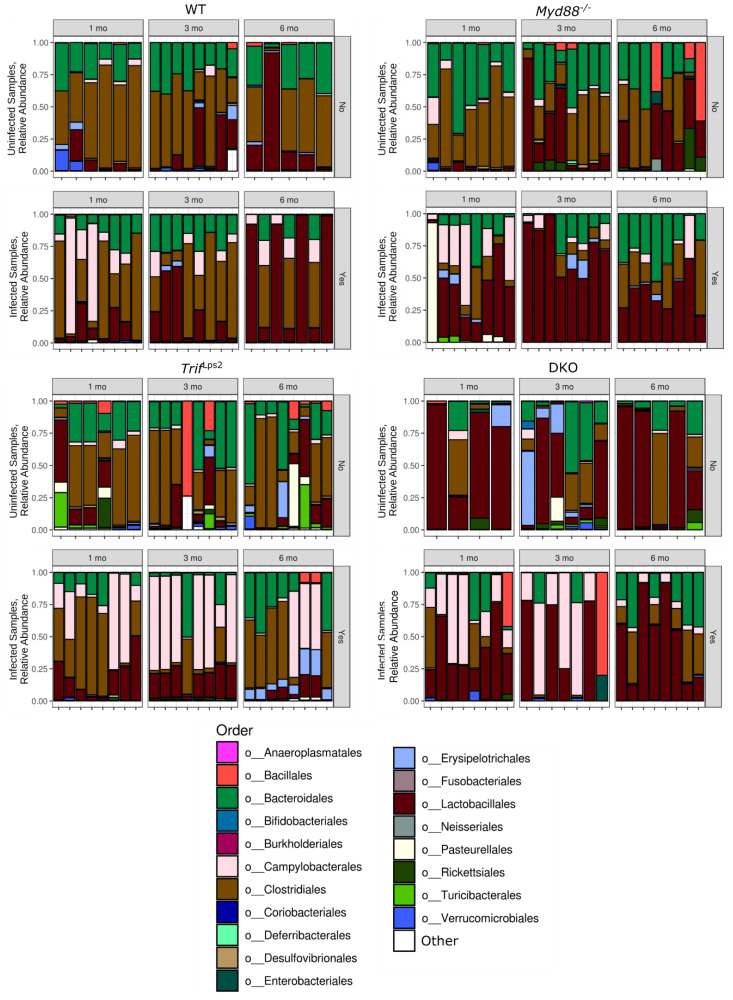
Relative abundance of different orders across four genotypes. Relative abundance of the top 15 orders from WT, *Myd88^−/−^, Trif*^Lps2^, and DKO genotypes. Remaining phyla are grouped into “Other”. Samples are grouped into *Helicobacter*-infected and non-*Helicobacter*-infected and divided by time point. Sequencing data were processed in QIIME2, then plotted in PhyloSeq.

We observed predominance of five orders across all genotypes—Bacteroidales, Campylobacterales, Clostridiales, Lactobacillales, Erysipelotrichales (Figure 3). The order Bacteroidales were observed across all genotypes with no overall significant difference. Clostridiales were present in all genotypes but less abundant in DKO mice. However, it was observed that levels of Clostridiales dropped significantly in *Myd88^−/−^* post infection as compared to uninfected samples especially at 3 months. Campylobacterales were observed more predominantly in infected samples in all four genotypes, as expected since *Helicobacter* is a member of the Campylobacterales order. However, the levels declined significantly at 3 and 6 months in *Myd88^−/−^* mice and at 6 months in DKO mice although Campylobacterales can be seen throughout all time points in *Trif*^Lps2^ infected mice. Lactobacillales were seen at highest relative abundance in *Myd88^−/−^* infected mice especially at 3 months followed by DKO infected mice, WT infected mice, and to a lesser extent *Trif*^Lps2^ mice (Figure 3). Erysipelotrichales were predominantly seen at 6 months in *Trif*^Lps2^ infected mice as compared to other genotypes. Apart from these orders, Rickettsiales were observed mainly in uninfected *Myd88^−/−^* and DKO mice as compared to *Trif*^Lps2^ and WT mice and were almost lost upon infection with *H. felis*. In addition, the order Bacillales were observed in uninfected samples of *Trif*^Lps2^, *Myd88^−/−^* and WT but not in DKO uninfected mice. However, they were observed in DKO post infection in one sample both at 1 month and 3 months and in two samples in *Trif*^Lps2^ at 6 months post infection with *H. felis*.

### 3.3. Association of Lactobacillales with Infection and Mouse Genotype

**Figure 4:**
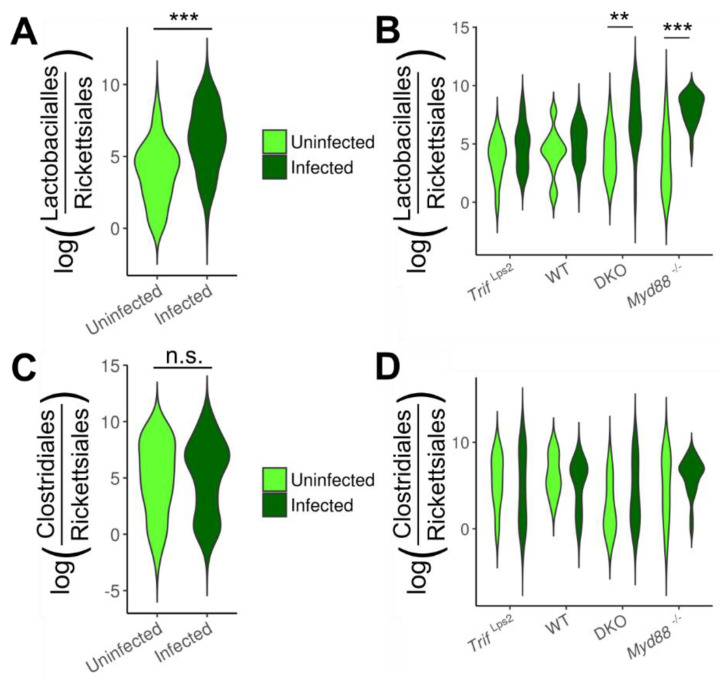
Log ratios between relevant orders across four genotypes. Log ratios of the Lactobacillales/Rickettsiales (**A**,**B**) and Clostridiales/Rickettsiales (**C**,**D**) relative abundance ratios between samples, months 1–6 combined. (**A**,**C**) show ratios by infection status, all genotypes combined. (**B**,**D**) show ratios by genotype and infection status. Log ratios were calculated and processed using Songbird and Qurro. Statistical significance was determined by analysis of variance (ANOVA) ** *p* < 0.01, *** *p* < 0.005).

Given the increase in Lactobacillales seen in infected mice by 16S rRNA amplicon sequencing (Figure 3) and the identification of *Lactobacillus* as a genus driving community dissimilarity (Figure 1C), we further investigated the relationship between changes in Lactobacillales and disease progression. We used the Songbird [20] and Qurro [21] tools to analyze changes in Lactobacillales between conditions. Songbird calculates log ratios of relative abundance ratios between two taxonomic units for each sample, a compositional analysis that accounts for absolute microbial abundance differences between samples [20]. Qurro can then be used to visualize and compare these differentials between samples [21]. This analysis revealed a highly significant increase in the log ratio of Lactobacillales/Rickettsiales (L/R) in infected communities compared to uninfected communities (Figure 4A). Analyzing this change within individual genotypes showed the log (L/R) ratio was significantly increased in infected *Myd88^−/−^* and DKO mice compared to uninfected (Figure 4B). The ratio was not significantly different between infected and uninfected communities in *Trif*^Lps2^ and WT mice. To confirm this finding, we repeated the analysis comparing Lactobacillales to other organisms present in all communities. Similar results were observed for log (Lactobacillales/Bacteroidales) (Figure S2A,B). No significant differences were seen in log ratios of the Clostridiales/Rickettsiales ratio (Figure 4C,D), and Clostridiales/Bacteroidales (Figure S2C,D) indicating the specificity of this finding to Lactobacillales. We therefore concluded that the order Lactobacillales is increased in infected communities in *Myd88^−/−^* and DKO, but not in WT and *Trif*^Lps2^ genotypes.

### 3.4. Lactobacillales Are Associated with Gastric Cancer Progression

**Figure 5:**
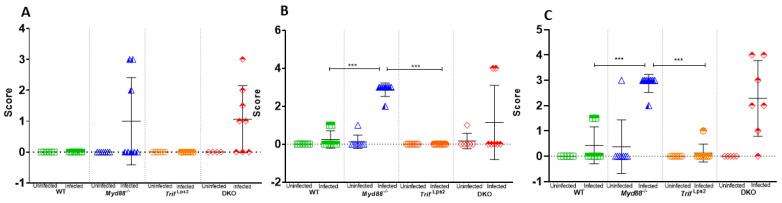
Histopathological scoring for mucous metaplasia. Following infection with *H. felis* for 1 month (**A**), 3 months (**B**) and 6 months (**C**), H&E-stained stomach sections from each mouse (WT and *Myd88*^−/−^, *Trif*^Lps2^and DKO) were evaluated for indications of pathology. Mucous metaplasia was scored by a blinded comparative pathologist according to the criteria described in Materials and Methods. A *p* value of 0.05 was considered statistically significant. (**A**) 1month post infection, *n* = 14 for WT, *n* = 15 for *Myd88*^−/−^, *n* = 14 for *Trif*^Lps2^, and *n* = 12 for DKO mice; (**B**) 3 months post infection, *n* = 16 for WT, *n* = 16 for Myd88^−/−^, *n* = 16 for Trif^Lps2^, *n* = 13 for DKO mice; (**C**) 6 months post infection, *n* = 12 for WT, *n* = 16 for Myd88^−/−^, *n* = 16 for Trif^Lps2^, *n* = 12 for DKO mice. Statistical significance was determined by Mann-Whitney test, *** *p* < 0.005).

**Figure 6:**
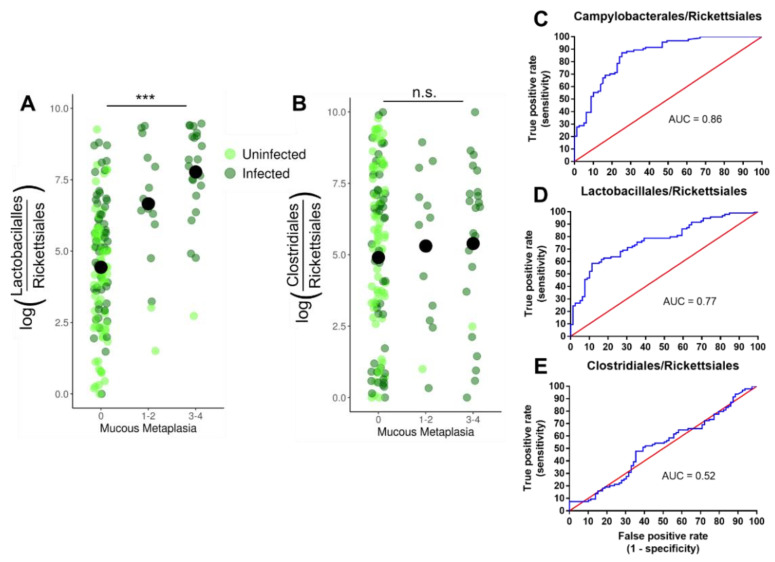
Predictive relationship between Lactobacillales and mucous metaplasia from *Helicobacter* infection. (**A**,**B**) Ordinal logistic regression analysis of log ratios of Lactobacillales/Rickettsiales (**A**), and Clostridiales/Rickettsiales (**B**) Relative abundance and gastric mucous histology score. The black circle marks the average of each category. Ordinal logistic regression was calculated using the polr command in R (*** *p* < 0.001). (**C**–**E**) ROC curve for log ratios of Campylobacterales, Lactobacillales, and Clostridiales to Rickettsiales. The blue line represents the performance of each ratio log fold-differential in predicting *Helicobacter* infection. The red line represents the result expected for a metric with a 50% chance of predicting infection. The area under the curve (AUC) value refers to the area under the blue line. Receiver operating characteristic (ROC) plots were constructed in Prism7 using log fold-differentials from Songbird and Qurro.

We next examined if there was an association between *Lactobacillales* levels and gastric disease progression in our fast-progressing model. Histological analysis revealed that *Myd88^−/−^* genotype displays the worst gastric disease following infection, followed by the DKO genotype. H&E stained stomach sections (Figure S3) from each mouse of each genotype (WT and *Myd88*^−/−^, *Trif*^Lps2^and DKO) were evaluated on basis of pathology and scored on scale of 0–4 (Figure 5). Since these genotypes also possessed the highest levels of Lactobacillales, we hypothesized higher Lactobacillales levels would be associated with higher mucous histology scores. Analysis of the log(L/R) ratio and the mucous histology score of each sample with ordinal logistic regression demonstrated that mice with a higher gastric Lactobacillales level had a significantly higher likelihood of a higher histology metaplasia score (Figure 6A). For the regression analysis, histology scores were grouped into categories of 0, 1–2, and 3–4 to increase statistical power. No association between Clostridiales levels and histology scores was observed (Figure 6B).

Given the association of Lactobacillales levels and mucous metaplasia, we examined the ability of Lactobacillales log ratios to predict if a mouse had been infected with *H. felis*. We constructed receiver operator characteristic (ROC) curves for log ratios of the orders Campylobacterales, Lactobacillales, and Clostridiales compared to Rickettsiales. Log (Lactobacillales/Rickettsiales) *ratios* predicted mouse infection status at a rate similar to log (Campylobacterales/Rickettsiales), the order containing *Helicobacter* and the logical “gold standard” for infection prediction (Figure 6C,D). In comparison, *log* (Clostridiales/Rickettsiales) *ratios* did not predict infection status at a rate better than random (Figure 6E). Together, these data indicate Lactobacillales correlates strongly with *Helicobacter* infection status and mucous histology score.

## 4. Discussion

Advancement in research on the gut microbiome has uncovered the existence of a diverse microbiome in the human gut in a delicate balance. These microbiota are vital for maintenance of human health and play an important role in energy metabolism, nutrient absorption and defense against pathogens [3,23,24,25]. However, if this balance is altered dysbiosis can lead to susceptibility to gastrointestinal pathogenesis and cancer [3]. The acidic environment of the stomach supports a smaller number of bacteria as compared to other parts of the gut, but dysbiosis due to various factors, such as genetic, environmental, or pathogen invasion can lead to gastric cancer [3]. The association of *Helicobacter* with gastric cancer has been well established, as it is characterized as a Type I carcinogen by the World Health Organization (WHO) [2]. Previous studies in insulin-gastrin (INS-GAS) mice have shown that *H. pylori* infections lead to an overall decrease in microbial density [26]. Our findings indicate there is also an overall decrease in microbiome diversity upon *H. felis* infections across all genotypes.

In this study we sampled the mouse stomach mucosal tissue instead of fecal samples to understand the changes specifically in the gastric microbiome in different genotypes, with respect to time as well as infection status. Analyzing stomach mucosal tissue provides a more accurate image of gastric microbial communities during gastric cancer progression than fecal samples. Moreover, as previously described *Myd88^−/−^* mice serve as a fast-progressing gastric cancer model, where gastric adenocarcinoma is reached within 6 months of infection with *Helicobacter* [8]. Therefore, analyzing gastric mucosa for the periods of 1 month, 3 months, and 6 months provides a clear picture of microbial diversity and composition fluctuations as disease progresses to gastric cancer.

In our study, Campylobacterales were observed in infected mice across all genotypes, which is expected as *Helicobacter* belongs to the order Campylobacterales. However, in *Myd88^−/−^*, our fast-progressing gastric cancer model, we observed a reduction in Campylobacterales abundance at 3 months and 6 months. This could be attributed to the fact that the advancement of gastric cancer lesions results in an increase in mucosal atrophy and decrease in acid secretion, potentially hindering *Helicobacter* colonization and facilitating an increase in abundance of other bacteria. In agreement with our study, patients with advanced atrophic gastritis have been found to have hypo-chlorohydric stomach mucosa and low abundance of *H. pylori*, with the gastric microbiome dominated by non-*Helicobacter* species [27,28]. A separate study by Basir et al. [29] revealed that increase in *H. pylori* colonization showed high correlation with severe chronic gastritis in human subjects. Similar correlations were observed by other groups [30,31] in their respective studies in gastric cancer patients. However, a study carried out on 273 human gastric biopsies revealed no relationship between *H pylori* density and chronic gastritis [32]. Thus, conflicting results have been observed when correlating *Helicobacter* density to severity of disease.

However, the low abundance of *H. pylori* appears to facilitate the dominance of other organisms of the microbiome. We observed an increase in Lactobacillales in infected *Myd88^−/−^* mice and DKO mice at 3 and 6 months. Previous studies on gastric cancer patients also showed increased abundance of Lactobacillales, supporting their possible role in gastric cancer progression [27,33] and in our case in a fast-progressing form of gastric cancer. Even though *Lactobacillus* species are utilized in probiotics and are commonly thought to be beneficial for the host, high levels of lactic acid can be detrimental in case of gastric cancer. Lactate can serve as a source of energy for tumor cells, which can lead to increased ATP production and promotion of inflammation [34,35,36,37,38]. Previous studies in INS-GAS mice have shown that mice harboring a complex microbiome develop gastric cancer in 7 months post infection with *H. pylori* as compared to *H. pylori* infections in germ free mice where development of gastric cancer is prolonged. In addition, supplementation of INS-GAS germ-free mice with a simplified microbiome of *Lactobacillus*, *Clostridium* and *Bacteroides* species was sufficient to promote development of gastric cancer [26]. This suggests the role of certain species in the gut microbiota in promoting gastric cancer progression.

Comparison of the gastric microbiome from *Myd88^−/−^* mice to WT, *Trif*^Lps2^ and DKO mice, we were able to intensively analyze how changes in Lactobacillales could be connected to gastric cancer development and progression. *Myd88*^−/−^ and DKO mice had significantly higher levels of Lactobacillales upon infection with *H. felis,* while WT and *Trif*^Lp2^ mice did not (Figure 4). *Myd88*^−/−^ and DKO mice also had significantly worse disease development than WT and *Trif*^Lp2^, as demonstrated by mucous metaplasia scores (Figure 5). These findings indicate a correlation between Lactobacillales levels and gastric cancer development that holds across genotypes. Ordinal logistic regression analysis and ROC curves (Figure 6) further demonstrate that the log-fold differential of Lactobacillales/Rickettsiales relative abundance ratio allows prediction of the infection status of a sample, irrespective of genotype. These data strongly indicate that Lactobacillales and gastric cancer progression are linked in these mouse models. A study carried out on gastric cancer patients from high-risk groups in Singapore and Malaysia revealed a high relative abundance of lactic acid-producing bacteria such as *Lactococcus* and *Lactobacillus*, and as well as oral cavity bacteria including *Fusobacterium*, *Veillonella*, *Leptotrichia*, *Haemophilus,* and *Campylobacter* [34]. This is in agreement with our findings that show a potential connection between gastric cancer and *Lactobacillus,* and further indicates that the *Myd88^−/−^* model of fast-progressing gastric cancer recapitulates a gastric microbiome change noted in human populations.

Recent studies in Taiwan, have reported that gastric cancer patients show increased colonization of *Clostridium* and *Fusobacterium* [39]. Other studies have shown that INS-GAS germ free mice develop gastric cancer when supplemented with *Lactobacillus* sp., *Clostridium* sp., and *Bacteroides* sp. [25]. In contrast to this, in our study the levels of Clostridiales significantly decreased in our fast progressing gastric cancer model as compared to other genotypes, suggesting that even though Clostridiales may have previously been shown to play a role in gastric cancer progression in other studies, it does not play a significant role in our fast-progressing gastric cancer model.

## 5. Conclusions

Our study suggests that differences in genotypes help define the stomach microbiome diversity as different genotypes have significantly dissimilar communities. However, the correlation between Lactobacillales levels and *Helicobacter*-induced gastric cancer progression holds across multiple genotypes. This sustained connection indicates Lactobacillales shares a close relationship with gastric cancer. The presence of Lactobacillales in the gastric microbiome needs to be further investigated to understand their probable role in the fast-progressing form of gastric cancer, as well as the stomach microbiome as a whole. Moreover, future studies need to be carried to observe any further alterations in gastric microbiome when cancer has fully developed helping us in understanding the role of the microbiome on disease outcome.

## Data Availability

The raw sequencing data presented in this study are openly available in the NCBI Sequence Read Archive (SRA) at accession number PRJNA685500.

## Supplementary Materials

The following are available online at https://www.mdpi.com/2076-2607/9/1/189/s1, Figure S1: Relative abundance of individual phyla, Figure S2: Log-fold differentials of Lactobacillales and Clostridiales with Bacteroidales, Figure S3: Histopathology of gastric disease induced by *H. felis* infection in different genotypes.

## Abbreviations

*H. pylori*	*Helicobacter pylori*
WHO	World Health Organization
*Myd88* ^−/−^	Myeloid differentiation primary response 88- deficient
*H. felis*	*Helicobacter felis*
TRIF	Toll/IL-1R (TIR) domain-containing adaptor-inducing interferon-β
*Trif* ^Lps2^	TRIF deficient
WT	Wild Type
DKO	double knockout
BHI	Brain Heart Infusion
QIIME2	Quantitative Insights into Microbial Ecology
TIR	Toll/interleukin-1 receptor
SEPP	SATé-enabled phylogenetic placement
OTU	Operational taxonomic unit
ROC	Receiver operating characteristic
FDR	False-discovery rate
INS-GAS	insulin-gastrin
IFN	interferon
