# Supplementary material for: Microbiome Signatures in a Fast- and Slow-Progressing Gastric Cancer Murine Model and Their Contribution to Gastric Carcinogenesis
Source: Microorganisms. 2021 Jan 17;9(1):189. doi: 10.3390/microorganisms9010189 (PMC7829848; doi:10.3390/microorganisms9010189)
Supplement: Supplementary file 1 [file microorganisms-09-00189-s001.zip › Supplementary material/Supplementary Figure legends.docx]

**Supplementary Figure Legends**

**Supplementary Figure S1. Relative abundance of individual phyla.** The relative abundance of the individual phyla *Firmicutes, Bacteroidetes, Proteobacteria, Verrucomicrobia,* and *Actionbacteria* are plotted. Samples are grouped into *Helicobacter*-infected and non- *Helicobacter*-infected and divided by genotype. Sequencing data were processed in QIIME2, then plotted in R with ggplot2.

**Supplementary Figure S2. Log-fold differentials of *Lactobacillales* and *Clostridiales* with *Bacteroidales*.** Log-fold differentials of *Lactobacillales/Bacteroidales* (A, B) and *Clostridiales/Bacteroidales* (C, D) ratios between samples. A and C show ratios by infection status, all genotypes combined. B and D show ratios by genotype infection and status. Log fold-differentials were calculated and processed using Songbird and Qurro. Statistical significance determined by ANOVA (* p<0.05, ** p<0.01, *** p<0.005).

**Supplementary Figure 3. Histopathology of gastric disease induced by *H. felis* infection in different genotypes.** Representative H&E stained sections of stomach from uninfected mice, infected mice at different time points 1, 3 and 6 months from all four genotypes Wild type (WT), MyD88 deficient (*Myd88^-/-^*), mice deficient in the TRIF, (*Trif^Lps2^*), and MyD88 and TRIF deficient (*Myd88^-/-^*/*Trif^Lps2^*, double knockout (DKO)).
