# Supplementary figures and images for: Microbiome Signatures in a Fast- and Slow-Progressing Gastric Cancer Murine Model and Their Contribution to Gastric Carcinogenesis
Source: Microorganisms. 2021 Jan 17;9(1):189. doi: 10.3390/microorganisms9010189 (PMC7829848; doi:10.3390/microorganisms9010189)

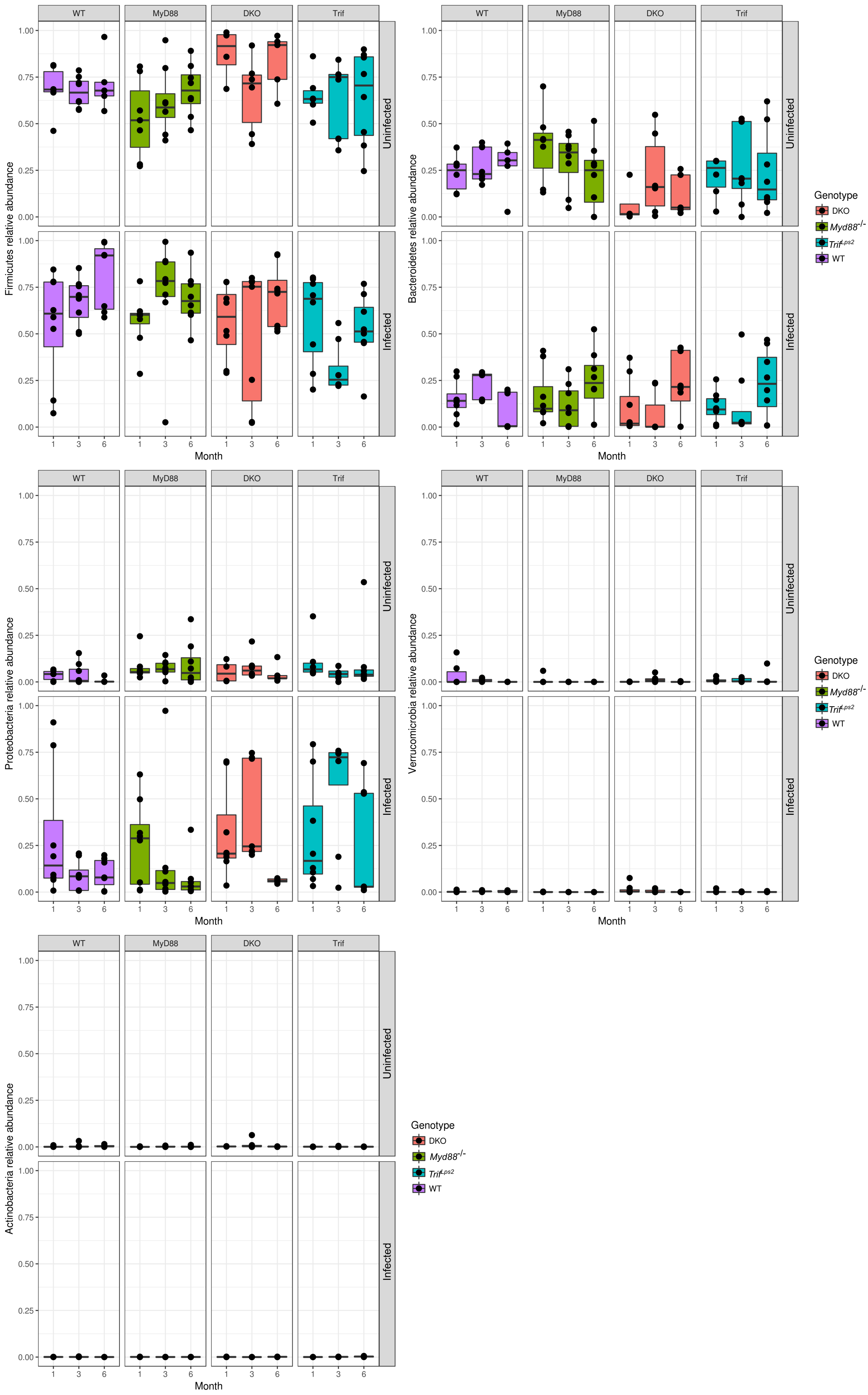

Supplement: Supplementary file 1 [file microorganisms-09-00189-s001.zip › Supplementary material/SupFigure1_12162020_v2.tif]

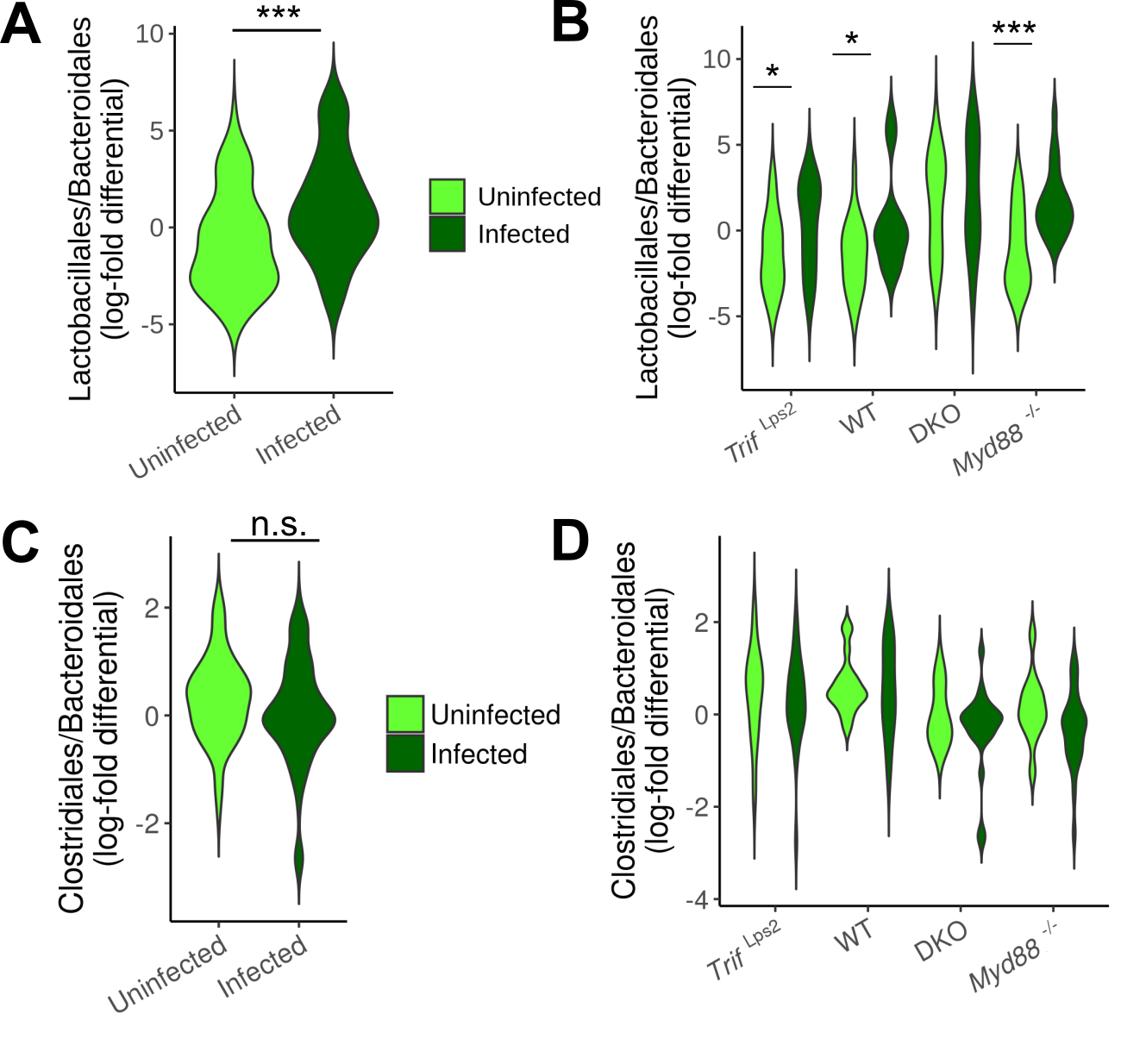

Supplement: Supplementary file 1 [file microorganisms-09-00189-s001.zip › Supplementary material/SuppFig2_12162020_v2.tif]

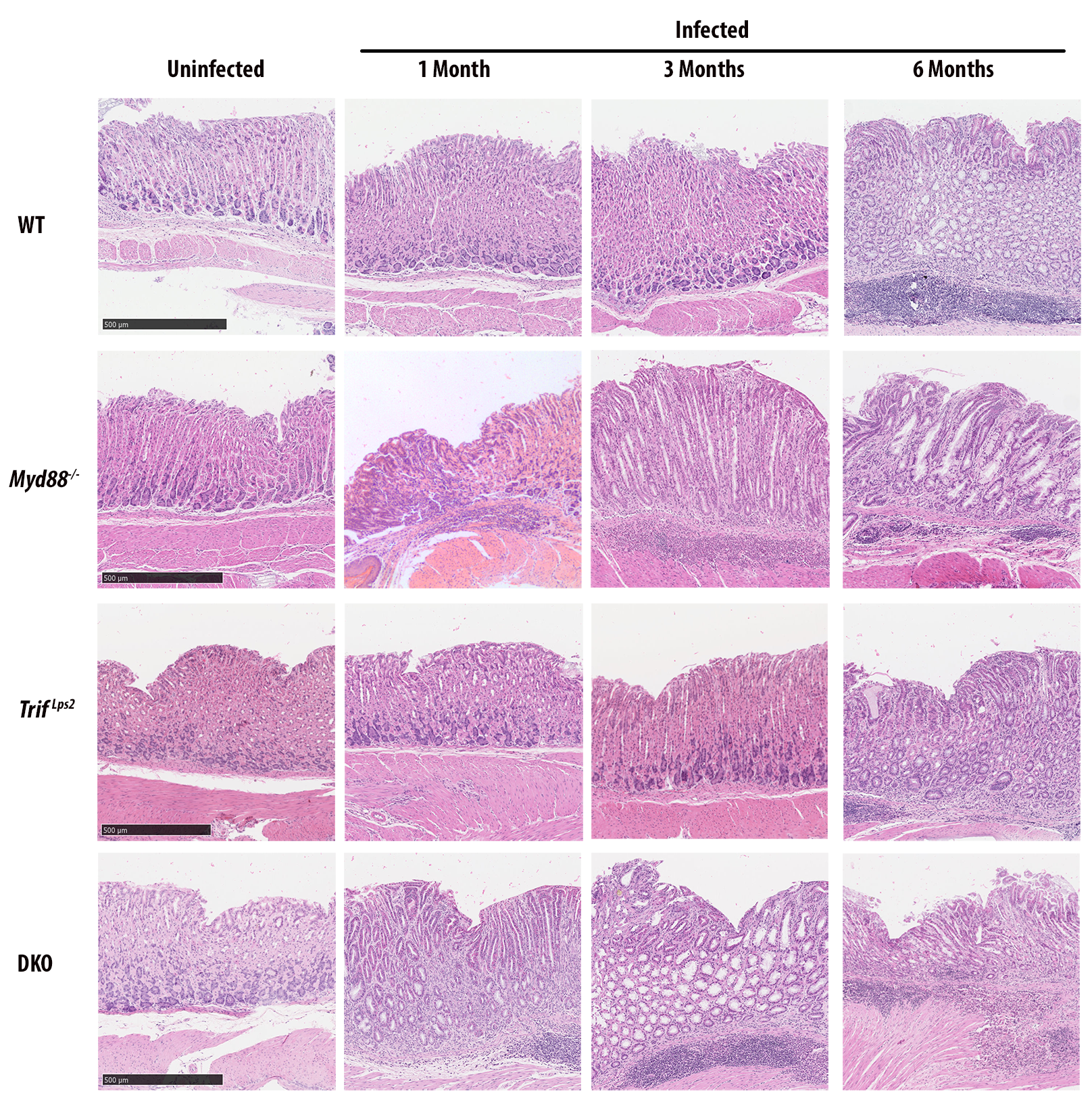

Supplement: Supplementary file 1 [file microorganisms-09-00189-s001.zip › Supplementary material/Supplementary Figure 3.tif]
